# Bridging gaps in traditional research training with iBiology Courses

**DOI:** 10.1371/journal.pbio.3002458

**Published:** 2024-01-11

**Authors:** Alexandra M. Schnoes, Noah H. Green, Thi A. Nguyen, Ronald D. Vale, Sarah S. Goodwin, Shannon L. Behrman

**Affiliations:** 1 Science Communication Lab, Berkeley, California, United States of America; 2 Howard Hughes Medical Institute, Janelia Research Campus, Ashburn, Virginia, United States of America

## Abstract

Training in technical and professional skills can be difficult to access for early career researchers. This Community Page highlights iBiology Courses, a free learning resource that provides training in navigating the research environment, becoming an effective researcher, and planning a research career.

## Teaser

iBiology Courses provide trainees with just-in-time learning resources to become effective researchers. These courses can help scientists build core research skills, plan their research projects and careers, and learn from scientists with diverse backgrounds.

Academic research training relies on a combination of traditional and experiential education to instruct early career researchers in technical and professional skills. The depth, breadth, and quality of graduate education depends on the graduate program and a trainee’s individual mentoring relationships. This variation means that many trainees will need additional training in researcher competencies to complement their research lab experience. However, many programs do not provide formal training for their students in competencies related to the process and culture of rigorous research science. This lack of comprehensive training can affect a trainee’s career efficacy, career intentions, scientific identity, and social and cultural capital [1].

In the past decade, the development of supplemental, online educational resources has greatly expanded to provide students with just-in-time (JIT) learning opportunities for skills, such as data analytics or programming [2]. However, these resources are not typically built for the early career scientist, nor do they specifically address the needs of life science trainees in developing research and professional skills, such as experimental design, research project planning, and scientist-to-scientist communication. Online courses are a JIT resource that can effectively fulfill these needs and provide an accessible learning environment that allows trainees to supplement the skills development they receive in their current research environment. Furthermore, providing these resources at no cost to trainees could address inequities in access and make a significant impact on the competency development and skill building for the larger research trainee population.

## iBiology Courses

iBiology Courses, designed by Science Communication Lab, is a no-cost resource that provides full courses to life science trainees on the process and practice of science, as well as related content for educators to use in their own classrooms and training programs. The online courses aim to guide undergraduate, graduate, and postdoctoral scholars through critical stages in scientific training by covering the following topics:

1. Planning Your Scientific Journey: how to plan a research project.
2. Let’s Experiment: how to design and execute experiments in the lab.
3. Share Your Research: how to give an effective scientific talk.
4. Business Concepts for Life Scientists: how to develop a research strategy (in academia and industry).

**Fig 1:**
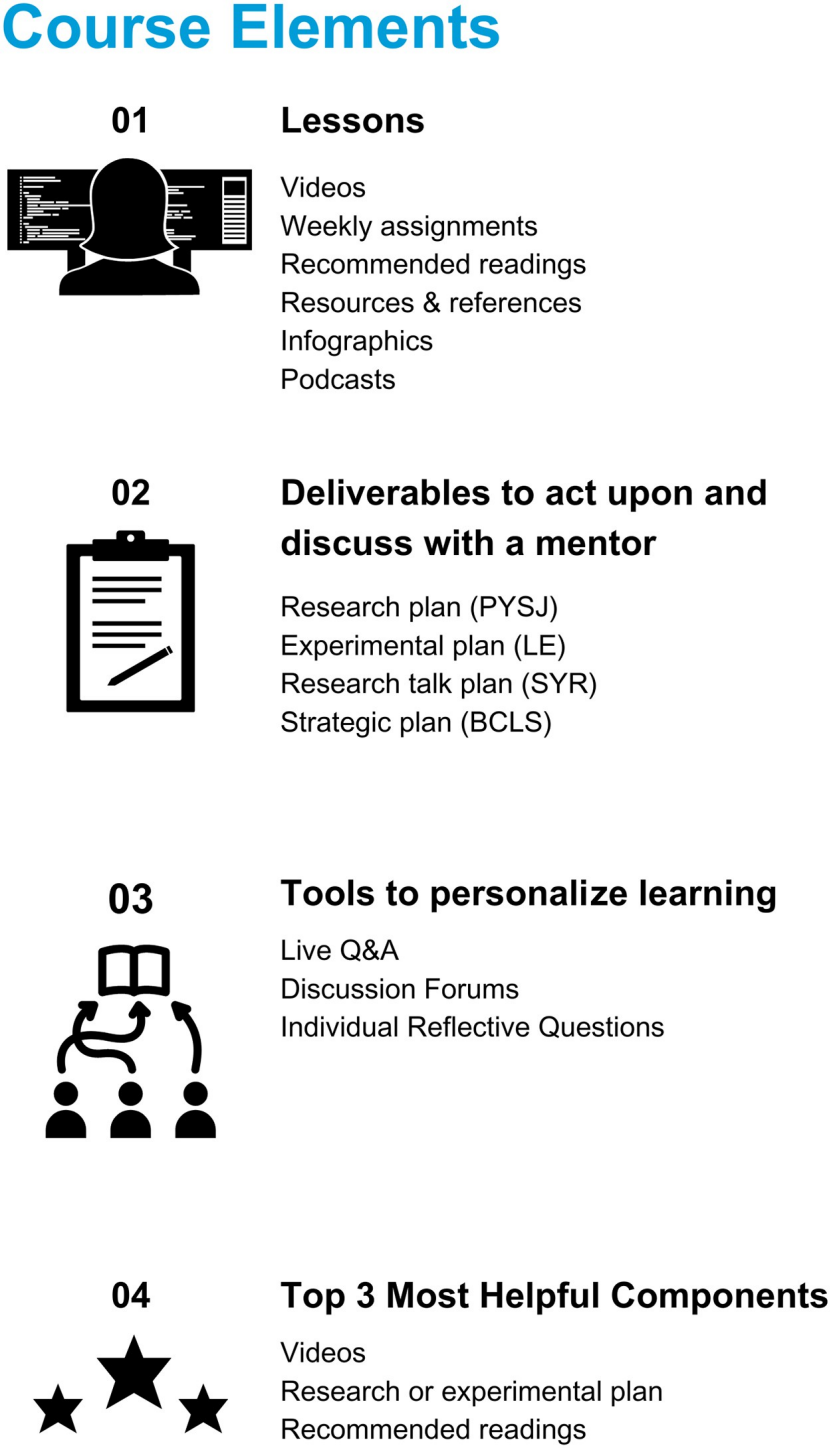
Elements of iBiology Courses that contribute to student learning and meaningful interactions with mentors. (01) The course lessons contain different modalities, such as videos and interactive prompts, designed to engage diverse learners and deepen their skills and knowledge. (02) By answering a series of reflective prompts throughout the course, participants create tangible plans that outline their goals, approaches, and anticipated outcomes relevant to the skills they want to develop in the lab. In each course, participants are directed to share their plans with mentors to receive feedback and guidance. (03) The courses include several ways for participants to personalize their own learning. (04) The most helpful learning components as identified by participants in surveys. BCLS, Business Concepts for Life Scientists; LE, Let’s Experiment; PYSJ, Planning Your Scientific Journey; SYR, Share Your Research.

Each course typically consists of 4 to 6 modules, and each module takes an estimated 2 to 3 hours of total work to complete. The modules are centered around specific learning outcomes and include a diverse combination of modalities, such as videos, podcasts, infographics, templates, reflective exercises/assignments, and recommended reading (Fig 1). The videos are multivoice, high quality, and include custom animations and slides to help visualize challenging concepts. They also feature interviews with scientists from diverse backgrounds and identities who share the nuances of science through concrete explanations, advice, stories, examples, and strategies. Each course conveys the crucial point that there is no one “right” way to do science, or one “ideal” background that results in good science. Learning from scientists from varied backgrounds has a positive impact on the development of scientific identity and resilience [3].

The course design ensures that learners apply what they learn to their own research projects and environments. Critical to each module, learners complete assignments primarily composed of reflective questions that are tied directly to their own research endeavors. Custom course infographics and templates make it easier to learn about complicated concepts and apply related skills. The variety of media makes the content fun, accessible, visual, relatable, and inclusive by providing different modalities of learning. Upon completion, students are presented with a compilation of their responses, which serve as a work plan they can use as an anchor point for moving forward, being productive, and getting feedback from their research mentors.

Roughly 19,000 individuals from around the world have enrolled in iBiology Courses since 2017. Students share benefits and career impacts through self-reporting in pre-course, post-course, and, in some cases, follow-up surveys, largely based on the Kirkpatrick framework on evaluating training programs (reaction, learning, and behavior) [4]. Participants reported learning gains in all courses, applied what they learned to their own research project/situation, and engaged more with their mentors in their research planning (Table 1).

**Table 1:** Benefits for participants who have taken iBiology Courses.

	Planning Your Scientific Journey, 2017	Let’s Experiment, 2018	Share Your Research, 2020	Business Concepts for Life Scientists, 2017
**The course “better prepared you for your upcoming scientific endeavors.”** [post-course survey]^a^	86% Yes, *N* = 163	88% Agreed or Strongly Agreed, *N* = 88	90% Agreed or Strongly Agreed, *N* = 201	*This question was not asked*
**The course helped you “implement a plan created during the course.”** [2–6-month follow-up survey]	88% Yes or Plan To, *N* = 93 (Yes = 60%, Plan To = 28%)	82% Yes or Plan To, *N* = 29 (Yes = 65%, Plan To = 17%)	84% Yes or Plan To, *N* = 108 (Yes = 54%, Plan To = 30%)	54% Agreed or Strongly Agreed, *N* = 24
**Participants “met with a mentor to discuss your research plan” or “gave a practice talk [SYR]” created during the course.** [2-month follow-up survey]	77% Yes or Plan To, *N* = 93 (Yes = 49%, Plan To = 28%)	63% Yes or Plan To, *N* = 29 (Yes = 54%, Plan To = 9%)	62% Yes or Plan To, *N* = 108 (Yes = 29%, Plan To = 33%)	*This question was not asked*
**The course “improved my process” for course-related skills.** [post-course or follow-up survey]	*This question was not asked*	93% Agreed, *N* = 88	89% Agreed, *N* = 201	58% Agreed, *N* = 24

^a^This study received IRB-approved exempt status through a review by the Committee on Human Research at the University of California, San Francisco (Study#: 15–17981). We received written consent from individuals participating in this study. Specifically, participants consented to publication of deidentified survey data by agreeing to the following statement: “If information from the study is published or presented at specific meetings, your name and other personal information will not be used.” The data underlying this table are raw data directly from survey questions. Our ethics approval allows the sharing of the deidentified raw data upon written request.

For each of the courses (Planning Your Scientific Journey, Let’s Experiment, Share Your Research, and Business Concepts for Life Scientists), participants created a plan (deliverable) for their research proposal, experiments, or presentation, depending on the course topic. Each plan was developed through step-by-step reflective prompts and the participants were asked to solicit feedback on the plan from a mentor. Many participants have reported referring to, implementing, and consulting with their mentors on their plans (Table 1).

## How can individuals use the courses?

iBiology Courses is open access and free for anyone to enroll and participate online. The course components are flexible and modular. Learners can interact with lessons, following their curiosity by browsing infographics, reading articles, watching videos, and participating in the online community through a forum. Learners can also create research experimental plans, research talk outlines, and career path plans by responding to self-reflective questions, and are encouraged to communicate their plans with their mentors. Upon course completion, learners receive both a certificate and a digital badge that they can add to LinkedIn. Student and postdoc groups can engage in courses independently or gather for “watch parties” to complete courses together. Each course also has a YouTube playlist, so it can be accessed from outside of the iBiology Courses platform.

## How can institutions use the courses?

iBiology Courses offer institutions valuable alternatives to creating time- and resource-intensive learning experiences on their own. The recorded expert interview portfolio of iBiology Courses can also provide enrichments to diversify an institution’s workshops, training courses, and course materials. Online courses can provide students with additional, supplemental instruction that is often requested by the graduate student body. Educators, faculty, and administrators supporting trainees have flexibility in implementation: They can assign one or multiple online modules or adopt hybrid formats where students watch a selection of videos and meet to discuss and reflect on relevant activities. iBiology Courses can also be implemented in partnership between graduate programs and university student affairs centers (e.g., career center, center for teaching and learning). When integrating the curriculum, educators can access lists of course learning objectives, videos, reflective assessments, and other related materials on the Educator Resources pages. Available under a Creative Commons license, these resources can be used by educators from around the world for their teaching and training needs.

## Conclusions

Online courses have several advantages, including flexibility, agency, and personalization. The learners participating in iBiology Courses documented growth in their foundation in critical skills, knowledge, and strategies to succeed in their own research endeavors. While there are limitations to self-reported data, and no online course can ever replace dedicated time working in the lab for building research-related skills, these online courses can serve as a supplementary resource to assist the learning process in addition to the training early career researchers already receive. As one learner who took Let’s Experiment stated, “This course touched on a lot of topics that I did not really consider before. Now… I am thinking about them a lot more and… that prepares me better for the moment those things become more relevant to my work.”
